# Identification of a virulence *tal* gene in the cotton pathogen, *Xanthomonas citri* pv. *malvacearum* strain Xss-V_2_–18

**DOI:** 10.1186/s12866-020-01783-x

**Published:** 2020-04-15

**Authors:** Fazal Haq, Shiwang Xie, Kunxuan Huang, Syed Mashab Ali Shah, Wenxiu Ma, Lulu Cai, Xiameng Xu, Zhengyin Xu, Sai Wang, Lifang Zou, Bo Zhu, Gongyou Chen

**Affiliations:** 1 School of Agriculture and Biology, Shanghai Jiao Tong University/Key Laboratory of Urban Agriculture by the Ministry of Agriculture, Shanghai, 200240 China; 2 State Key laboratory of Microbial Metabolism, School of life Science and Biotechnology, Shanghai Jiao Tong University, Shanghai, 200240 China

**Keywords:** Bacterial blight of cotton, *Xanthomonas citri* pv. *malvacearum*, Transcription-activator-like effector, Virulence

## Abstract

**Background:**

Bacterial blight of cotton (BBC), which is caused by the bacterium *Xanthomonas citri pv. malvacearum* (*Xcm*), is a destructive disease in cotton. Transcription activator-like effectors (TALEs), encoded by *tal*-genes, play critical roles in the pathogenesis of xanthomonads. Characterized strains of cotton pathogenic *Xcm* harbor 8–12 different *tal* genes and only one of them is functionally decoded. Further identification of novel *tal* genes in *Xcm* strains with virulence contributions are prerequisite to decipher the *Xcm*-cotton interactions.

**Results:**

In this study, we identified six *tal* genes in Xss-V_2_–18, a highly-virulent strain of *Xcm* from China, and assessed their role in BBC. RFLP-based Southern hybridization assays indicated that Xss-V_2_–18 harbors the six *tal* genes on a plasmid. The plasmid-encoded *tal* genes were isolated by cloning *Bam*HI fragments and screening clones by colony hybridization. The *tal* genes were sequenced by inserting a Tn*5* transposon in the DNA encoding the central repeat region (CRR) of each *tal* gene. *Xcm* TALome evolutionary relationship based on TALEs CRR revealed relatedness of Xss-V_2_–18 to MSCT1 and MS14003 from the United States. However, Tal2 of Xss-V_2_–18 differs at two repeat variable diresidues (RVDs) from Tal6 and Tal26 in MSCT1 and MS14003, respectively, inferred functional dissimilarity. The suicide vector pKMS1 was then used to construct *tal* deletion mutants in *Xcm* Xss-V_2_–18. The mutants were evaluated for pathogenicity in cotton based on symptomology and growth *in planta*. Four mutants showed attenuated virulence and all contained mutations in *tal2*. One *tal2* mutant designated M2 was further investigated in complementation assays. When *tal2* was introduced into *Xcm* M2 and expressed in *trans*, the mutant was complemented for both symptoms and growth *in planta*, thus indicating that *tal2* functions as a virulence factor in *Xcm* Xss-V_2_–18.

**Conclusions:**

Overall, the results demonstrated that Tal2 is a major pathogenicity factor in *Xcm* strain Xss-V_2_–18 that contributes significantly in BBC. This study provides a foundation for future efforts aimed at identifying susceptibility genes in cotton that are targeted by Tal2.

## Background

Cotton (*Gossypium* spp.) is an economically-important crop worldwide and is a significant source of fiber, feed, oil and biofuel [1]. The primary cotton production areas are located in the southern United States (USA), Central America, western Africa, and central and eastern Asia. According to the 2017/18 world ranking, China leads the world in cotton production followed by India, the USA and Pakistan [2]. *Gossypium* spp. contains over 50 species, including *G. arboreum*, *G. herbaceum*, *G. hirsutum* and *G. barbadense*. *G. arboretum* and *G. herbaceum* are diploid (2n = 26), whereas *G. hirsutum* and *G. barbadense* are tetraploid (4n = 52) [3, 4]. *G. hirsutum* is the predominant species and produces with 90% of the world’s cotton fiber production [5]. This species is impacted by a devastating bacterial disease known as bacterial blight of cotton (BBC), which is caused by *Xanthomonas citri* pv. *malvacearum*. The first detailed description of BBC was reported in the USA [6]. However, this disease currently occurs in all cotton production areas and causes significant yield losses (5–35%) either by injury to the plant or direct damage to the boll [7].

*Xcm* is able to infects all above-ground parts of cotton at any developmental stage starting with seedlings [8]. Typical BBC symptoms include cotyledon/seedling blight, angular leaf spots, water-soaked lesions, black arm of petioles and stems, boll rot and boll shedding [8, 9]. The main virulence factors that contribute to the pathogenicity and adaptation of bacterial pathogens include exopolysaccharides, lipopolysaccharides, adhesins, protein secretion systems, siderophores, quorum sensing, biofilms, chemotactic sensors and degradative enzymes [10–13]. Particularly, type III secreted effector (T3SE) proteins play an important role in bacterial pathogenicity [10–12, 14] and have been identified in *Xanthomonas* spp. [14–20]. One of the most studied groups of T3SEs are the transcriptional-activator like (*tal*) effector (TALE) proteins [21–28].

TALE proteins, functionally resemble eukaryotic transcription factors, are localized to the host plant nucleus where they bind to specific promoter sequences known as effector-binding elements (EBEs), thus regulating host gene expression [29–31]. TALEs belong to the *avrBs3*/*pthA* gene family [26], which is highly conserved among different *Xanthomonas* spp. TALEs contain an N-terminal T3S signal domain, a central repeat region (CRR), C-terminal nuclear localization signals (NLS), and an acid activation domain (AD) [30, 31]. CRRs contain tandem repeats of 33–35 amino acids that differ only at residues 12 and 13; these are designated repeat variable di-residues (RVDs) and determine the specificity of DNA binding [30–32]. TALE-mediated activation of EBEs can induce host susceptibility (*S*) or resistance (*R*) genes [29, 30]. For example, the TALEs PthXo1 and PthXo2 from *X. oryzae* pv. *oryzae* (*Xoo*) were shown to enhance the expression of rice genes *OsSWEET11* and *OsSWEET13*, which are required for susceptibility to bacterial leaf blight [33, 34]. However, rice cultivars were resistant to *Xoo* when they contained *OsSWEET11* and *OsSWEET13* alleles lacking PthXo1 and PthXo2 EBEs [35–37]. Recently, a new rice *S* gene (*OsERF#123*) was shown to be targeted by TalB in African strains of *Xoo* [38]. Other examples of TALEs include AvrBs3 that targets the pepper resistance gene *Bs3* and AvrXa10, AvrXa23 and AvrXa27 that interact with rice *R* genes *Xa10*, *Xa23* and *Xa27*, respectively [29, 39–41]. Recently, Cai et al. [21] reported that Tal7 from *Xoo* binds and activates the expression of the rice gene *Os09g29100*, an interaction that suppresses *avrXa7-Xa7-*mediated resistance in rice. A number of truncated TALEs (truncTALEs) and interfering TALEs (iTALEs) have also been reported in *Xoo* that function as suppressors of *Xa1*-mediated defense in rice [42, 43].

Resistance to *Xcm* has been identified primarily in *G. hirsutum*. The genetic nature of resistance to BBC was first revealed in 1939, and efforts to breed cotton plants for resistance ensued shortly thereafter [44]. About 20 major *R* genes or polygene complexes (*B* genes) participate in resistance to BBC in cotton [7, 8]. Based on their virulence phenotype in differential cotton hosts, *Xcm* strains have been classified into 22 races that are named 1–22 [7]. Race 18 is the most common variant and was first isolated in 1973 [45, 46]. In some cases, the outcome of interactions between *Xcm* strains and differential cotton varieties is dependent on the *avrBs3*/*pthA* gene family in *Xcm*, which indicates that *Xcm*-cotton interactions follow the gene-for-gene model for host plant resistance [7, 10, 47, 48].

The number and diversity of *tal* effector genes varies among different species, pathovars and strains of *Xanthomonas*. For example, *Xoo* strains harbor 8–26 TALEs [49–53], *Xoc* strains contain 19–28 [49, 54, 55], *Xtt* strains contains 5–12 [56], *Xtu* strains contains 7–8 [56–58] and *Xcm* strains harbor 8–12 genes encoding *tal* effectors [27, 46, 59]. Some *Xanthomonas* spp. lack *tal* effector genes, such as *X*. *citri* pv. *raphani* strain 756C [54]. To date, at least 20 TALEs have been cloned and characterized from *Xcm* strains [25, 26, 28, 48]. Among these, Avrb6 was the first *Xcm* TALE shown to be important for virulence [25]. Cox et al. [27] demonstrated that Avrb6 induced the expression of the cotton *S* gene, *GhSWEET10*, thus enhancing bacterial virulence and adaptation to the host.

The aim of the current study is to identify a novel virulent *tal*-gene encoding TALE protein in a highly virulent cotton pathogen, *Xcm* strain Xss-V_2_–18 (from China).

## Results

### TALEs of Xss-V_2_–18

**Fig. 1:**
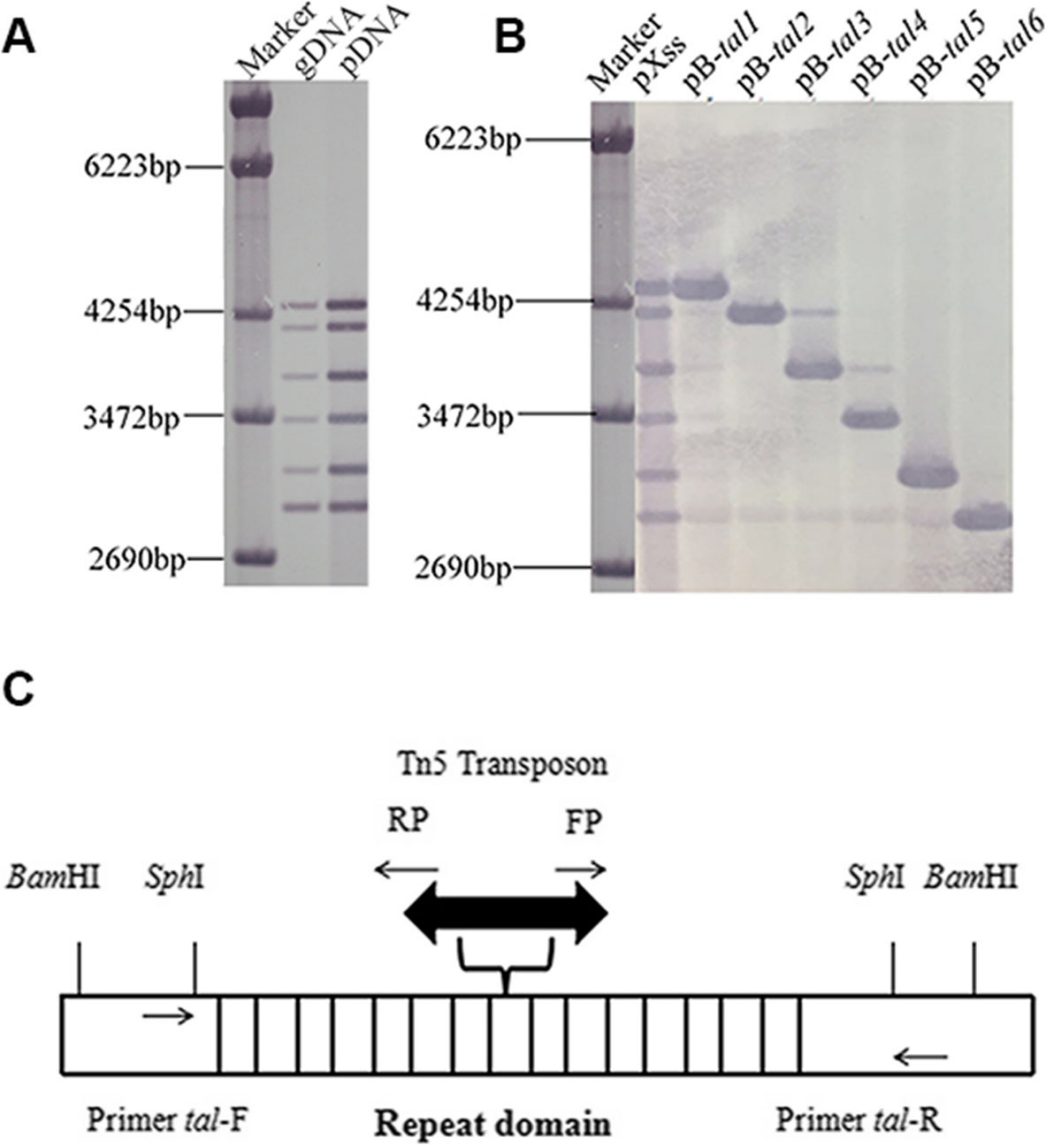
Southern blotting, and Isolation and sequencing of Xss-V_2_–18 *tal-*genes. **a** Southern blot analysis of *Bam*HI-digested genomic (gDNA) and plasmid DNA (pDNA) of *Xcm* strain Xss-V_2_–18. A 2.9-kb *Sph*I fragment of *pthXo1* (from *Xoo*) was labeled with digoxygenin (DIG) and used as a probe to detect *tal* genes in *Xcm* Xss-V_2_–18. **b** Plasmid DNA of Xss-V_2_–18 was digested with *Bam*HI, and fragments were gel-purified and ligated into *Bam*HI-digested and CIP-treated pBluescript II SK(−). Southern blot analysis was performed by the using internal *Sph*I fragment of *pthXo1* as a probe to confirm each clone (pB-*tal1* – pB-*tal6*). **c** Schematic diagram of strategy used to sequence *tal* genes. After cloning into pBluescript II SK(−), the EZ-Tn5™ < KAN-2 > Tnp Transposome™ Kit was used to insert Tn*5* into each *tal* gene. Clones with Tn*5* insertions in the middle of the CRR were selected by *Sph*I digestion and sequenced using primer pairs tal-F/RP and FP/tal-R

Restriction fragment length polymorphism (RFLP) analysis was conducted to estimate the number and size of *tal* genes in *Xcm* Xss-V_2_–18. Since most *tal* genes retain two *Bam*HI sites, *Xcm* Xss-V_2_–18 plasmid and genomic DNAs were digested with *Bam*HI and analyzed by Southern blotting as described above. Six bands hybridized to the probe in *Bam*HI-digested genomic and plasmid DNA, indicating that Xss-V_2_–18 contained six plasmid-encoded *tal* genes (Fig. 1a).

**Fig. 2:**
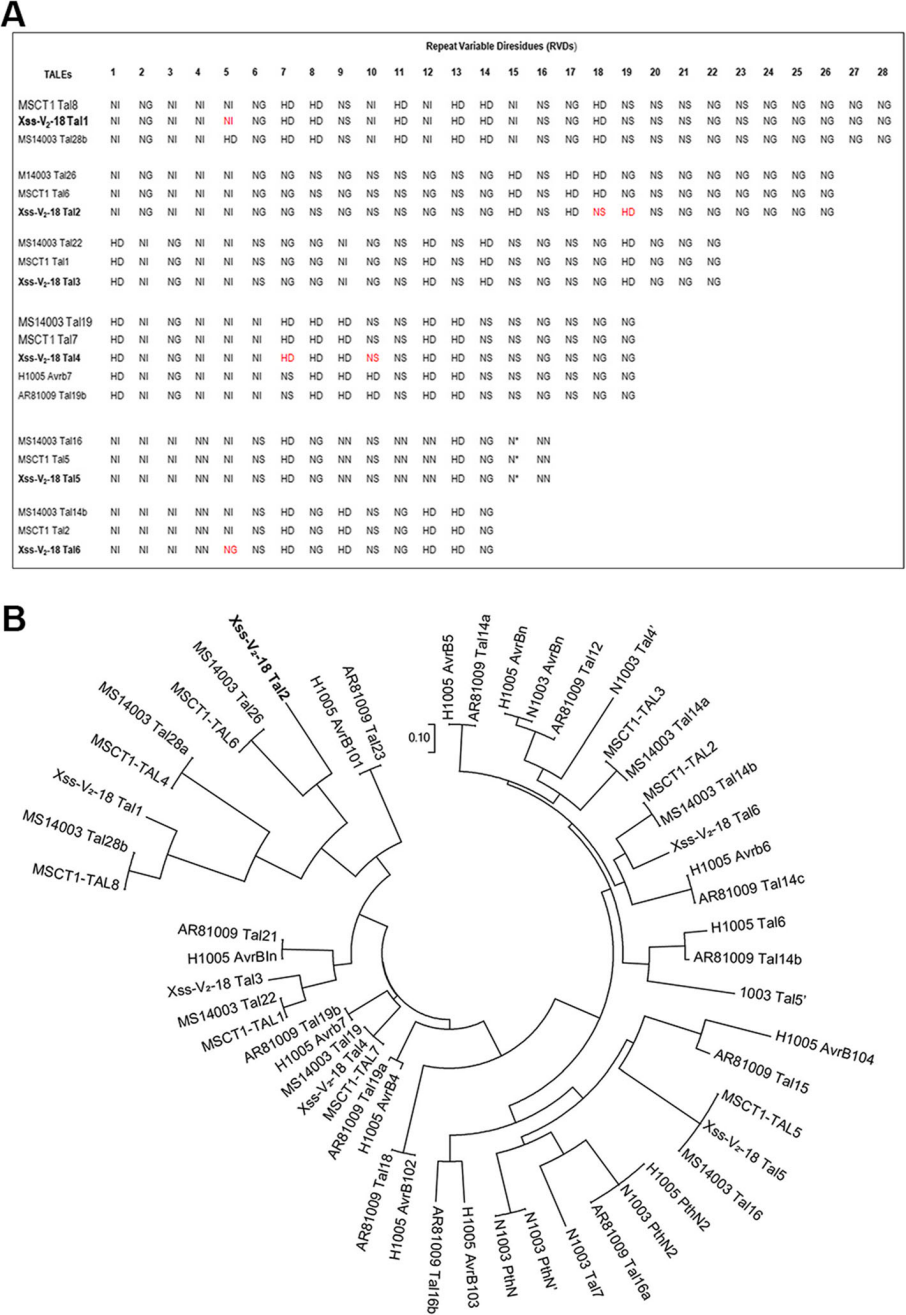
Alignment of TALE RVDs and TALEs Phylogeny. **a** Alignment of TALE RVDs from *Xcm* strains Xss-V_2_*–18,* MSCT1, H1005, MS14003 and AR81009 with AnnoTALE (version 1.4.1). Letters in red font indicate RVDs that differ between the two strains. The asterisk represents a missing amino acid residue **b** Construction of phylogenetic tree based on central repeat amino acid *sequences of TALEs. A set of 53* TAL effector sequences from *6* different *Xcm* strains were used to construct tree with DisTAL program using default parameters. TALEs were classified into 6 major groups and 33 sub-groups showing the relationship of *Xcm* Xss-V_2_–18 to other *Xcm* strains published previously. Tal2 of Xss-V_2_–18, TAL6 of MCST and Tal26 (M26) of MS14003 fall in same group. Scale is shown below the tree

The six *tal* genes were cloned in pBluescript as *Bam*HI fragments, giving rise to pB-*tal1*, pB-*tal2*, pB-*tal3*, pB-*tal4*, pB-*tal5* and pB-*tal6* (Fig. 1b) and confirmed by colony hybridization and sequence analysis. To obtain the complete DNA sequence of each *tal* gene, we inserted the Tn*5* transposon into the CRR region and used primer sets tal-F/RP and FP/tal-R to obtain the sequences (Fig. 1c). The *tal* gene sequences have been deposited in GenBank under the following accession numbers: MK654746 (*tal1*), MK654747 (*tal2*), MK654748 (*tal3*), MK654749 (*tal4*), MK654750 (*tal5*) and MK654751 (*tal6*). Each *tal* gene encodes various numbers of RVDs, which are tandemly arranged and encoded within 102-bp direct repeats. There were 27.5, 102-bp repeat units in *tal1*, 25.5 in *tal2*, 21.5 in *tal3*, 18.5 in *tal4*, 15.5 in *tal5* and 13.5 in *tal6* (Fig. 2a).

To better understand the features of Xss-V_2_–18 TALEs, we compared them with TALEs in *Xcm* strains MSCT1, H1005, N1003, MS14003 and AR81009 [27, 46, 59]. Phylogenetic tree of TALEs from *Xcm* strains were constructed by aligning TALE-CRR with DisTAL v1.1. All 53 TALEs (Xss-V_2_–18 = 6, MSCT1 = 8, H1005 = 12, N1003 = 9, MS14003 = 8 and AR81009 = 12) were classified into 6 major groups and 33 sub-groups. Tal2 of Xss-V_2_–18, TAL6 of MCST and Tal26 of MS14003 fall in same group (Fig. 2b).

Nearly identical RVD sequences were observed for the six TALEs in Xss-V_2_*–18,* MSCT1, H1005, MS14003 and AR81009 (Fig. 2a). Differences of two RVDs between Tal2 of Xss-V2–18 and TAL6 of MSCT1, Tal26 of MS14003 indicate that they are functionally different from each other and may target a different EBE. The predicted theoretical EBE box for Tal2, Tal6 and Tal26 of Xss-V_2_–18, MSCT1 and MS14003, respectively, are mentioned in Fig. S1. RVDs in *Xcm* strains included NI, NG, NS, HD and NN; the latter RVD was absent in Tal1, Tal2, Tal3 and Tal4.

### Xss-V_2_–18 *tal* deletion mutants

**Fig. 3:**
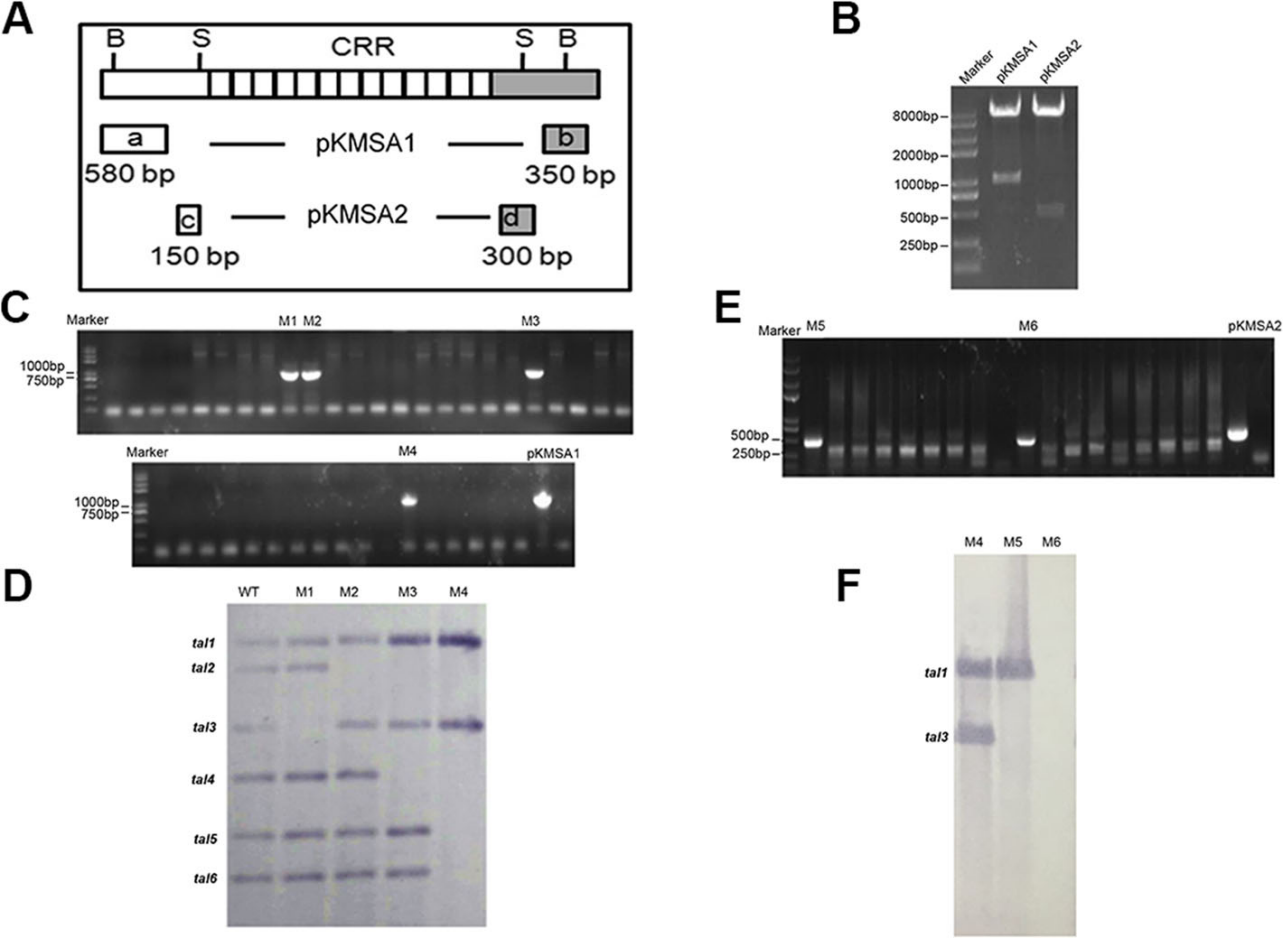
Deletion mutagenesis of Xss-V_2_–18 *tal* genes. **a** Schematic diagram of suicide plasmids pKMSA1 and pKMSA2. Fragments *a* (580 bp) and *b* (350 bp) were amplified on the left and right sides of the CRR, respectively, and cloned as a fused fragment in pKMSA1. Fragments *c* (580 bp) and *d* (150 bp) were amplified on the left and right sides of the CRR, respectively, and cloned as a fused fragment in pKMSA2. Constructs pKMSA1 and pKMSA2 were introduced into *Xcm* strain Xss- V_2_–18 by electroporation, and deletion of the CRR region was conducted as described in Methods. **b** Confirmation of 930- and 450- bp inserts in pKMSA1 and pKMSA2, respectively, by digestion with *Xba*I and *Sma*I. **c** PCR analysis of 41 putative mutants with primers pKMSA1-5F and pKMSA1-3R. A 930-bp fragment was amplified in M1, M2, M3, and M4, indicating that these four mutants underwent a homologous recombination and potential deletion of the CRR region. pKMSA1 was included as a control. **d** Southern hybridization analysis of Xss-V_2_–18 and mutant strains M1-M4. Plasmid DNA of WT Xss-V_2_–18 and mutants were isolated and digested with *Bam*HI. The internal *Sph*I fragment of *pthXo1* (from *Xoo*) was used as a hybridization probe to detect *tal* genes. **e** PCR screening for putative mutants using primers pKMSA2-5F and pKMSA2-3R. pKMSA2 was included and used as a positive control. **f** Southern hybridization analysis of mutant M4 (used for second round of mutagenesis), M5 and M6. Plasmid DNA of M4, M5 and M6 were isolated and digested with *Bam*HI, and the internal *Sph*I fragment of *pthXo1* was used a hybridization probe to detect *tal* genes

To assess the role of *tal* genes in the virulence of Xss-V_2_–18, we generated *tal* deletion mutants by homologous recombination using the suicide vector pKMS1 [60]. Fragments *a* (580 bp) and *b* (350 bp) were amplified on the left and right sides of DNA encoding the CRR, respectively, and cloned as a fused fragment in pKMSA1 (Fig. 3a, b). Construct pKMSA1 was introduced into *Xcm* Xss-V_2_–18; after homologous recombination, 41 putative mutants were selected for PCR amplification using primers pKMSA1-5F/pKMSA1-3R (Table S1). Four putative mutants designated M1, M2, M3 and M4 contained a 930-bp PCR product, which is consistent with the size of the insert in pKMSA1 (Table 1, Fig. 3c). Southern hybridization indicated that one or more *tal* genes were deleted in the four mutants (Fig. 3d). M1 and M2 were lacking *tal3* and *tal2*, respectively, M3 was missing *tal2* and *tal4*, and M4 lacked *tal2*, *tal4*, *tal5*, and *tal6*. These results indicated that four *tal* loci underwent homologous exchange via pKMSA1, and copies of the plasmid pKMSA1 functioned to delete multiple *tal* genes simultaneously in M3 and M4.

**Table 1:** List of strains and plasmids used in this study

Strain or plasmid	Relevant characteristics	Source
*Escherichia coli*		
DH5α	*F*^*−*^*φ80 lacZΔM15Δ(lacZYA-argF)U169 deoR recA1 endA1 hsdR17(rk-, mk+) phoA supE44 λ- thi-1 gyrA96 relA1*	Clontech
*X. citri* pv. *malvacearum*		
MSCT1	Wild-type, causes bacterial blight of cotton	[59]
XcmH1005	Spontaneous Rif^r^ derivative of XcmN	[27]
XcmH1003	Sp^r^, Rif^r^ derivative of XcmN	[27]
Xcc049	Wild-type *Xcc* strain, used for construction of pKMSA1/A2	This lab
Xss-V_2_–18	Wild-type, causes bacterial blight of cotton	Hainan University, China
M1	*tal3* deletion mutant of Xss-V_2_–18	This study
M2	*tal2* deletion mutant of Xss-V_2_–18	This study
M3	*tal2 tal4* deletion mutant of Xss-V_2_–18	This study
M4	*tal2 tal4 tal5 tal6* deletion mutant of Xss-V_2_–18	This study
M5	*tal2 tal3 tal4 tal5 tal6* deletion mutant of Xss-V_2_–18	This study
M6	*tal-*free mutant of Xss-V_2_–18	This study
**Plasmids**		
pBluescript II SK(−)	Ap^r^, phagemid, pUC derivative	Lab collection
pMD18-T	Ap^r^, pUC18 derivative, TA cloning vector, 2692 bp	TaKaRa
pKMS1	Km^r^, *sacB mob lacZ oriV*, 6475 bp	[60]
pHM1	Broad-spectrum cosmid vector, *cos*, *parA*, *IncW*, Sp^r^	[61]
pKMSA1	pKMS1 containing a 930-bp *Xba*I/ *Sma*I fragment; insert contains a fusion of *a* and *b* fragments that encode the N- and C-terminal sides of the *tal* CRR; Km^r^	This study
pKMSA2	pKMS1 containing a 450-bp *Xba*I/ *Sma*I fragment; insert contains a fusion of the *c* and *d* fragments that encode the N- and C-terminal sides of the *tal* CRR; Km^r^	This study
pB-*tal1*	pBluescriptIISK(−) containing *tal1* of Xss-V_2_–18	This study
pB-*tal2*	pBluescript II SK(−) containing *tal2* of Xss-V_2_–18	This study
pB-*tal3*	pBluescript II SK(−) containing *tal3* of Xss-V_2_–18	This study
pB-*tal4*	pBluescript II SK(−) containing *tal4* of Xss-V_2_–18	This study
pB-*tal5*	pBluescript II SK(−) containing *tal5* of Xss-V_2_–18	This study
pB-*tal6*	pBluescript II SK(−) containing *tal6* of Xss-V_2_–18	This study
pZWavrXa7	*avrXa7* in pBluescript II KS+, contains FLAG epitope immediately downstream of the second *Sph*I site in the C-terminus of AvrXa7, Ap^r^	[62]
pZW-*tal2*	*Sph*I fragment of *tal2* in pZWavrXa7, Ap^r^	This study
pHZW-*tal2*	pHM1 fused with pZW-*tal2* at *Hin*dIII, *lacZ* promoter upstream of *tal2*, Ap^r^, Sp^r^	This study

A second round of deletion mutagenesis was conducted with plasmid pKMSA2, which contains a fusion of fragments *c* (150 bp) and *d* (300 bp) on the left and right sides of the DNA encoding the CRR, respectively (Fig. 3a). Construct pKMSA2 was used to generate new deletions in the M4 mutant, and potential new mutants were analyzed by PCR with primer pairs pKMSA2-5F/pKMSA2-3R (Table S1). Two mutants designated M5 and M6 contained a 450-bp PCR product that is consistent with the size of the insert in pKMSA2 (Fig. 3e). In addition to *tal2*, *tal4*, *tal5*, and *tal6*, Southern hybridization indicated that mutant M5 contained a deletion in *tal3.* M6 was lacking both *tal1* and *tal3* (Fig. 3f); thus, M6 lacked all six *tal* genes and can be considered a *tal*-free mutant of Xss-V_2_–18.

### Virulence assays

**Fig. 4:**
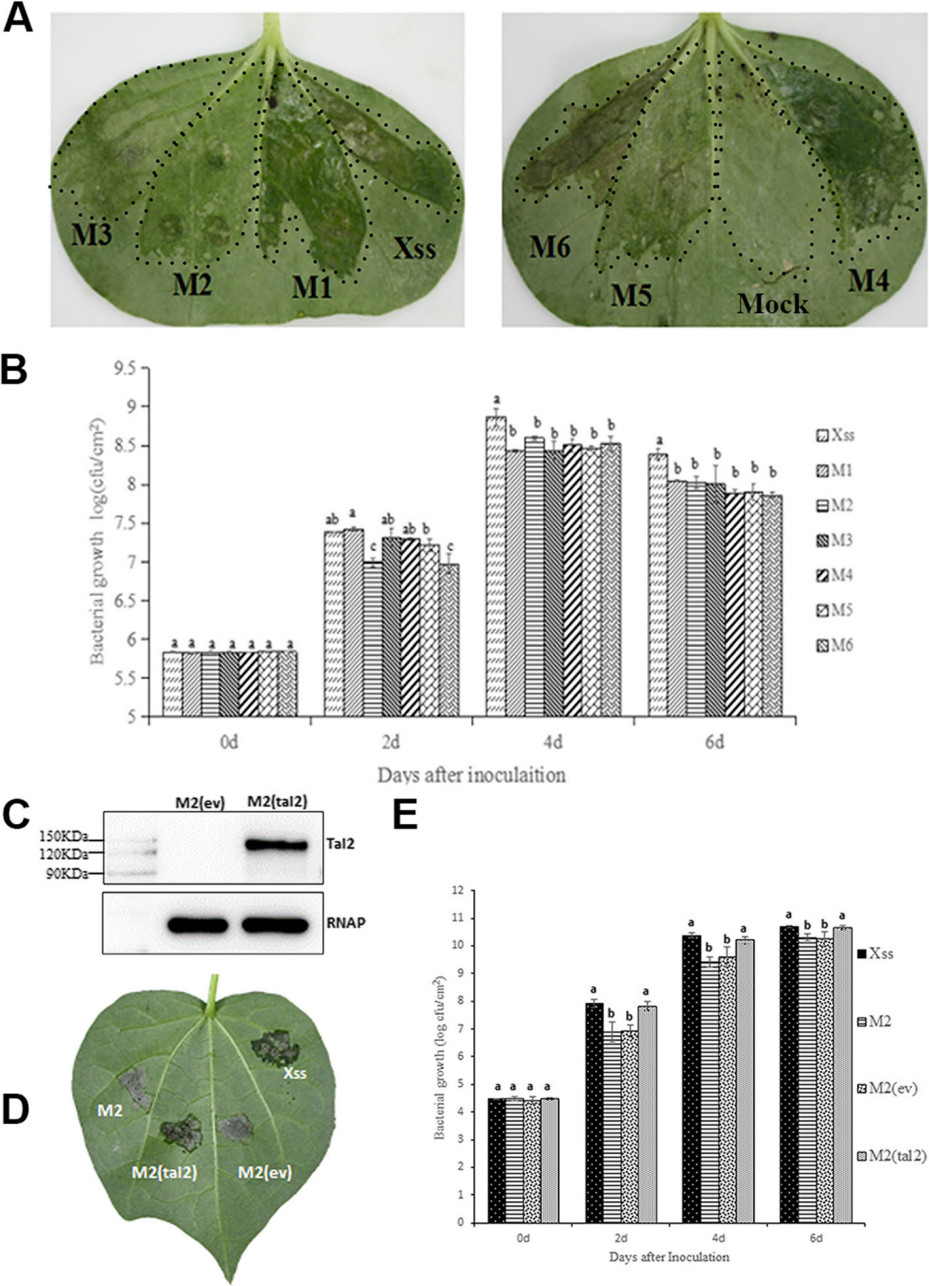
Tal2 contribution to virulence of Xss-V_2_–18 on cotton variety TM-1. **a** Phenotypes of the mutant strains relative to wild-type Xss-V_2_–18. Wild-type (WT) and mutant strains were inoculated to the lower surface of cotton leaves (two-week-old plants) using a needleless syringe. Infiltration with simply 10 mM MgCl2 served as a mock. Phenotypes were observed 3–5 days post-inoculation. **b***In planta* growth of WT Xss-V_2_–18 and mutants. Growth was measured at 0, 2, 4, and 6 days post-inoculation. Error bars represent means and standard deviations (means ± SD), and columns labeled with different letters represent significant differences (*P* < 0.05). **c** Western blot analysis of TALE production in *Xcm* M2. Plasmid pHZW-*tal2* was transferred into *Xcm* M2 by electroporation. Production of TALE was analyzed by western blotting using an anti-FLAG primary antibody (see Methods). RNA polymerase subunit alpha (RNAP) from *E. coli*, was used as a loading control. **d** Symptoms in cotton leaves inoculated with Xss-V_2_–18, mutant M2, M2 containing empty vector and M2 containing *tal2 in trans*. Bacterial strains were inoculated to cotton leaves using a needleless syringe, and phenotypes were observed within 5–7 days post-inoculation. **e***In planta* growth of the WT Xss-V_2_–18, mutant M2 and complemented strain. Growth was measured at 0, 2, 4, and 6 days post-inoculation. Error bars represent means and standard deviations (means ± SD), and columns labeled with different letters represent significant differences (*P* < 0.05)

Xss-V_2_–18 and mutants M1-M6 were inoculated into cotton leaves and phenotypes were observed 3–5 days post-inoculation (Fig. 4a). Xss-V_2_–18, M1, and M4 produced substantial water-soaked lesions in the inoculation sites; however, water-soaking was reduced in leaves inoculated with M2, M3, and M5 (Fig. 4a). In contrast, the region inoculated with the *tal*-free mutant M6 showed cell death and necrosis (Fig. 4a) signifying that the loss of *tal* genes affect the virulence of Xss-V_2_–18. On the second day post-inoculation, the populations of the M2 and M6 mutants were significantly lower than Xss-V_2_–18, M1, M3, M4 and M5 (Fig. 4b). On days 4 and 6 post-inoculation, the growth of Xss-V_2_–18 was significantly higher than mutants M1-M6 with no significant difference among the mutants. These results indicated that some of the *tal* genes are involved in Xss-V_2_–18 virulence, and the absence of selected *tal* genes impacted growth of the pathogen *in planta*.

Mutant M2, which lacks *tal2,* exhibited reduced symptomology and bacterial growth when compared to wild-type Xss-V_2_–18 (Fig. 4a, b). Based on these observations, we speculated that *tal2* might be involved in virulence; this was addressed by constructing pHZW-*tal2* (Table 1) for complementation analysis. The pHZW-*tal2* construct was introduced into *Xcm* M2, and the empty vector (ev, pHM1) was used as a negative control. Western blot analysis indicated that the Tal2 protein was produced in *Xcm* M2 (Fig. 4c). The wild-type Xss-V_2_–18, mutant M2, M2(ev), and M2(*tal2*) were inoculated into cotton leaves; phenotypes were observed at 5–7 days post-inoculation (Fig. 4d), and bacterial growth was measured at 0, 2, 4, and 6 days post-inoculation (Fig. 4e). Both water-soaking and bacterial growth *in planta* were restored to wild-type levels in *Xcm* M2 containing pHZW-*tal2* (Fig. 4d, e). Based on results shown in Fig. 4, we conclude that Tal2 is major virulence factor in Xss-V_2_–18.

## Discussion

Until recently, BBC has been effectively controlled using classical *R* genes [63–65]; however, in 2011 the disease re-emerged with a vengeance [46]. A known virulence factors, transcription activator-like effectors (TALEs), in *Xcm* are important for BBC. In previous studies, 8–12 *tal* genes were reported in *Xcm* [26–28, 48, 59]. Some *Xcm tal* genes, notably *avrB101*, *avrB102* and *avrBln*, are known to cause an hypersensitive response (HR) on cotton [28], whereas *avrb6* elicits water-soaking [48]. In this study, RFLP-based Southern hybridizations indicated that the highly-virulent *Xcm* strain Xss-V_2_–18, which was originally isolated from Hainan, China, harbors six plasmid-borne *tal* genes (Fig. 1). In the genus *Xanthomonas*, the location and number of *tal* genes varies among species, pathovars and strains [55, 66]. For example, strains of *X. oryzae* pv. *oryzicola* (*Xoc*) encode over 250 chromosomally-borne *tal* genes [55]; however, plasmid-encoded *tal* genes are common in other *Xanthomonas* spp. Examples include the *tal* genes in *X. citri* pv. *citri*, *X. citri* pv. *aurantifolii* and *X. axonopodis* pv. *manihotis,* which were identified on plasmids pXAC66, pXcB and pXam46, respectively [67–69]. Feyter and Gabriel [28] and Showmaker et al. [59] reported the existence of plasmid-borne *tal* genes in *Xcm* strains XcmH and MSCT1, respectively. A draft genome sequence of the *Xanthomonas translucens* pv. *cerealis* strain CFBP 2541 also indicate a plasmid borne *tal*-gene [70].

The presence of highly repetitive sequences in *tal* genes complicates efforts to obtain their nucleotide sequence; therefore, we used a Tn*5* insertion method as a sequencing strategy. This sequencing strategy for *tal*-genes was also used by others previously [21, 71]. Normally the number of repeats in *tal* genes varies between 1.5 and 33.5, and each repeat encodes 33–34 amino acids that vary only at positions 12 and 13 (RVDs) [30]. In *Xcm* Xss-V_2_–18, we identified 27.5, 25.5, 21.5, 18.5, 15.5 and 13.5 tandemly arranged 102-bp direct repeats (encoding 34 amino acids) in *tal1*, *tal2*, *tal3*, *tal4*, *tal5* and *tal6*, respectively. In order to understand how *Xcm* TALome differ from each other within and between strains, DisTAL and AnnoTALE were used to characterized [50, 72]. *Xcm* encodes very diverse TAL effectors that were classified exclusively into 6 major groups and 33 sub-groups. TALE phylogenetic tree of *Xcm* strains showed that Tal2 of Xss-V_2_–18, TAL6 of MCST and Tal26 (M26) of MS14003 fall in same group. Furthermore, RVDs based analysis showed that the six TALEs in Xss-V_2_–18 were identical or nearly identical to plasmid-borne TALEs in *Xcm* MSCT1, MS14003, H1005 and AR81009 which suggests that these genes may have been horizontally transferred [67, 73, 74]. The number and location of *tal* genes varied in the six *Xcm* strains; MSCT1 possess eight (seven plasmid-borne) [59], XcmH1005 has 12 (six plasmid- and six chromosomally-encoded) [27], XcmN1003 has nine (four plasmid-encoded) [27], MS14003 has 8 (7 plasmid-encoded) [46], AR81009 has 12 (six plasmid-encode) [46] and Xss-V_2_–18 has six plasmid-encoded *tal* genes (Figs. 1, 2). The variation in number, location and RVD sequence in *Xcm* TALEs could be important for maintaining virulence in cotton cultivars grown in different geographical regions.

To assess the role of *tal* genes in Xss-V_2_–18, we generated deletions in Xss-V_2_–18 by homologous recombination with pKMS1 [60], which was previously used to generate deletion mutants in the rice pathogen, *Xoc* [75–77]. This is the first report where pKMS1 was used to generate *tal* deletion mutants in *Xcm*, and the basic strategy was to replace the CRR (encoded by 102-bp repeat units) with up-and downstream fragments flanking the *tal* genes. Using construct pKMSA1, we obtained four mutants; M1 and M2 lacked *tal3* and *tal2*, M3 had deletions in *tal2* and *tal4*, and M4 lacked *tal2*, *tal4*, *tal5* and *tal6*. We speculate that *tal5* and *tal6* might be located in the same gene cluster. The second knockout was obtained using pKMSA2 where up- and downstream flanking fragments (homology arms) were located closer to the CRR. Mutant M4 was used as a parental strain for the deletions generated with pKMSA2, and we recovered two new mutants designated M5 and M6. In addition to *tal2, tal4, tal5* and *tal6*, mutant M5 also lacks *tal3*, whereas M6 contains deletions in all six *tal* genes (Fig. 3). The symptoms induced by M2, M3, M5 and M6 were significantly reduced relative to the wild-type, thus indicating that one or more *tal* genes contribute to symptom development in Xss-V_2_–18. Mutants M2, M3, M5 and M6 all lack the *tal2* gene; thus the potential contribution of *tal2* to symptom development was further investigated. Expression of *tal2 in trans* restored symptoms and growth *in planta* to the M2 mutant, thus confirming that Tal2 is a virulence factor (Fig. 4). Although the TALE repertoire of *Xcm* Xss-V_2_–18, MSCT1, MS14003, H1005 and AR81009 is somewhat identical, Tal2 of Xss-V_2_–18 differs at two repeat variable diresidues (RVDs) from Tal6 in MSCT1 and Tal26 in MS14003, inferred functional dissimilarity.

TALEs functionally resemble eukaryotic transcription factors that target and regulate the expression of host genes by binding to their promoter sequences. TALE-triggered susceptibility has been well-studied, and the contribution of TALEs to virulence has been evaluated in many *Xanthomonas* spp. [21–23, 27, 57, 78–81]. For example, the TALEs PthXo1 and PthXo2 from *Xoo* were shown to enhance the expression of rice genes *OsSWEET11* and *OsSWEET13*, which are required for susceptibility to bacterial leaf blight in rice [33, 34]. However, rice cultivars were resistant to *Xoo* when they contained *OsSWEET11* and *OsSWEET13* alleles lacking PthXo1 and PthXo2 EBEs [35–37]. A recent study by Peng et al. [82] reported that Tal8 from *Xtu* target and induce the expression of host gene *Ta-NCED-5BS*, encode enzyme required for rate-limiting step in ABA biosynthesis, to promote disease susceptibility. In another new study, Wu et al. [83] shown that TAL-effector Brg11 from *Ralstonia solanacearum* enhance the expression of 5́-truncated *ADC* (*arginine decarboxylase*) transcripts that subvert translational control and thereby inhibit competing pathogens. In *Xcm*, Avrb6 was the first TALE shown to be important for virulence [25]. Recently, the *Xcm* effector Avrb6 was shown to target and induce the expression of the cotton *S* gene, *GhSWEET10*, thus enhancing virulence and promoting disease [27]. The present study provides an important foundation for identifying potential *S* genes that interact with Tal2, which will ultimately help us develop better control strategies for BBC.

## Conclusions

In this study, we identified genes encoding TALEs in the highly-virulent *Xcm* strain, Xss-V_2_–18 (from China), and assessed TALE roles in BBC. We found that Xss-V_2_–18 encodes six plasmid-borne *tal* genes. Knockout mutagenesis of Xss-V_2_–18 *tal* genes and complementation analysis demonstrated that Tal2 is required for full virulence of Xss-V_2_–18 on cotton. The identification of the Tal2 target in cotton will ultimately provide new avenues for developing BBC-resistant varieties.

## Methods

### Bacterial strains, growth conditions, and plasmids

The bacterial strains and plasmids used in this study are listed in Table 1. *Escherichia coli* strains were grown in Luria-Bertani (LB) medium (5 g yeast extract, 10 g NaCl, 10 g tryptone/L) or LB with agar at 37 °C. *Xcm* strains were grown in nutrient broth (NB) (1 g yeast extract, 3 g beef extract, 5 g polypeptone and 10 g sucrose/L) or NB with agar at 28 °C. *Xcm* transformants containing the first crossover event were grown on NAN (nutrient agar without sucrose) or NBN (NAN without agar) medium. For the second crossover event, transformants were plated on NAS agar (NAN with 10% sucrose) [60]. When appropriate, antibiotics were added at the following concentrations (μg/mL): ampicillin, 100; kanamycin, 20; spectinomycin, 25; and rifampicin, 50. The pH of both solid and liquid media was adjusted to 7.0–7.2.

### DNA preparation

Total genomic DNA of Xss-V_2_–18 was isolated using the Bacterial Genomic DNA Extraction Kit (TaKaRa, China). The isolated gDNA pellet was re-suspended in double-distilled water. Bacterial plasmid DNA was isolated using the Plasmid Miniprep Kit (Omega, USA). The quality and quantity of genomic DNA and plasmid DNA were checked with NanoDrop spectrophotometer (Eppendorf). Routine plasmids isolation from *E. coli* was carried out by using the plasmid DNA Mini Kit (GBS Biotechnology, China).

### Isolating, cloning and sequencing of Xss-V_2_–18 *tal* genes

The isolation and cloning of *tal* genes from *Xcm* strain Xcc-V_2_–18 followed a previously described procedure [21, 71, 79, 84] with minor modifications. Plasmid DNA and genomic DNA (50 μg) were isolated from *Xcm*, digested with *Bam*HI, and separated on 1.2% agarose gels. Specific *tal* DNA fragments were then gel-purified and ligated into pBluescriptII SK(−) that was digested with *Bam*HI and treated with calf intestinal phosphatase (CIP). The ligated products were introduced into competent *E. coli* cells by the heat shock method according to the manufacturer’s protocol (Bio-Rad, USA). The successful cloning of *tal* genes in pBluescript II was validated by restriction digestion, colony hybridization and sequence analysis.

The repeat units in *tal* genes complicate abilities to sequence the genes using conventional approaches. Thus, after cloning into pBluescript II SK(−), we used the EZ-Tn5™ < KAN-2 > Tnp Transposome™ Kit to insert Tn*5* into each *tal* gene as recommended by the manufacturer (Epicentre, Madison, WI). Clones with Tn*5* insertions in the middle of the repeat region were selected by *Sph*I digestion and sequenced using primers pair tal-F/RP and FP/tal-R (Table S1).

### TALEs phylogenetic tree construction and RVDs comparison

For TALEs phylogeny, available genome sequences of *Xcm* strains MSCT1, H1005, N1003, MS14003 and AR81009 were obtained from the NCBI. *TALE* genes were predicted and analyzed in each genome using AnnoTALE v1.4.1 [50]. DisTAL v1.1 were used to align and classify TALEs based on their central repeat region [72].

For the TALE RVDs analysis, we used AnnoTALE version 1.4.1. The TALEs are grouped into classes based on the RVDs that shows possible functional and evolutionary relationship [50, 85].

### Construction of Xss-V_2_–18 *tal* deletion mutants

The *tal* genes in *Xcm* Xss-V_2_–18 were deleted by homologous recombination using the suicide vector pKMS1 [60]. The 5*′* and 3*′* fragments that flank the CRR repeat in *tal* genes are conserved [66] and were used as sites for homologous recombination. The left- and right-flanking fragments of each *tal* gene were PCR-amplified using genomic DNA of *Xcc* strain Xcc049 (Table 1) as the template, and ligated into the MCS of pKMS1 [4], resulting in constructs pKMSA1 and pKMSA2, respectively. The new constructs were verified by restriction digestion and sequence analysis (TaKaRa, China). Constructs pKMSA1 and pKMSA2 were introduced into *Xcm* strain Xss-V_2_–18 by electroporation; cells were then plated on NAN medium supplemented with kanamycin and incubated at 28 °C for 4 days. Single colonies were then cultured in NBN broth at 28 °C to OD_600_ ≤ 0.2 (~ 3 × 10^8^ cells/mL), inoculated to NAS agar medium, and incubated for 2 days at 28 °C. Single colonies that grew on NAS were then transferred to NA and NA containing kanamycin. Colonies that grew on NA, but not on NA^Km^, were selected as potential deletion mutants. The mutants were then analyzed by Southern blot hybridization and PCR with primer pairs pKMSA1-5F/pKMSA1-3R and pKMSA2-5F/pKMSA2-3R (Table S1).

### Southern hybridizations

*Xcm* plasmid and genomic DNA were extracted as described above. After *Bam*HI digestion, DNA was separated on 1.2% agarose gels and then transferred onto Hybond N^+^ nylon membranes (Roche, Germany). The 2898-bp internal *Sph*I fragment of *pthXo1* (GenBank accession no: AY495676) from *Xoo* [86] was labeled with digoxigenin (DIG) and used as a hybridization probe to detect the *tal* genes. Probe labeling and Southern blotting were performed using the DIG Probe Synthesis Kit as recommended by the manufacturer’s instructions (Roche, Sweden).

### Virulence assays

Cotton cultivar TM-1 (*G. hirsutum*) was used in this study. Plants were grown in a greenhouse at 23 °C with a 12-h light/dark photoperiod and ~ 80% RH. Two-week-old plants were used in virulence assays. Single colonies of *Xcm* were inoculated to 4 mL NB and cultured overnight at 28 °C. Bacterial cells were harvested by centrifugation (5000 rpm, 3 min); pellets were washed twice in sterile 10 mM MgCl_2_ and then re-suspended in 10 mM MgCl_2_ buffer to OD_600_ = 0.1 (~ 2 × 10^8^ cells/mL). The suspensions were inoculated to the abaxial surface of leaves by infiltration with a sterile needleless syringe. Inoculation with simply 10 mM MgCl_2_ buffer served as a mock. Leaf phenotypes were examined 4–5 days after inoculation. Three independent plants were used, and the experiments were repeated three times with similar results. For the quantification of bacterial growth in cotton, triplicate leaf samples (1cm^2^ in diameter) were collected for each inoculated strain and washed with 70% ethanol and double-distilled water (ddw). Samples were macerated in 1 mL ddw and incubated for 30 min at room temperature. Serial dilutions were then plated on NB agar medium with appropriate antibiotics for colony counts. The experiment was repeated three times, and the significant differences were determined by using student’s *t*-test.

### Expression of *tal2* gene in *Xcm* M2

The plasmid pZWavrXa7 (supplied by Dr. Bing Yang) was used to construct the plasmid for expression of *tal2* in Xss-V_2_–18 strain. Plasmid pZWavrXa7 contains a FLAG-tag epitope immediately downstream of the second *Sph*I site in the C-terminus of AvrXa7. The central *Sph*I fragment of *avrXa7* was replaced with the *Sph*I fragment of Xss-V_2_–18 *tal2* gene to generate pZW-*tal2* (Table 1). The recombinant plasmid was then fused with broad-host-range vector pHM1 at the *Hin*dIII site giving rise to pHZW-*tal2*. The constructs were transformed into *Xcm* M2 (∆*tal*2 strain, see below) by electroporation (2.5 kv, 4 ms).

The expression of *tal2* in M2 was confirmed by western blotting with flag-labelled antisera. Briefly, the M2 strain containing pHZW-*tal2* was cultured in NB to the logarithmic phase and harvested by centrifugation. The pellets were washed twice, and re-suspended in 1X PBS buffer to OD_600_ = 1.0 (~ 3 × 10^9^ cells/mL). SDS loading buffer (5X) was added to the bacterial suspensions and boiled in a water bath for 10 min. Proteins were separated on 8% SDS-PAGE gels and transferred to polyvinylidene difluoride membranes for immunoblotting using anti-FLAG (TransGene, Beijing, China) as the primary antibody. Primary antibodies were detected using goat anti-mouse IgG (H + L) (TransGen) and visualized with the EasySee Western Kit (TransGen). *E. coli* RNA polymerase subunit α (RNAP) was used as a loading control.

## Supplementary information

**Additional file 1: Table S1.** Primers used in this study. **Figure S1.** Predicted theoretical target site logo. **(A)** Target site logo for Tal2 of Xss-V_2_-18. **(B)** Target site logo for Tal6 of MSCT1 and Tal26 of MS14003. Based on TALgetter (Galaxy v1.1 http://galaxy.informatik.uni-halle.de/)

## Data Availability

All the dataset generated or analyzed during this study are included in this published article. The nucleotide sequences have been deposited in GenBank under the following accession numbers (MK654746-MK654751). The plasmids are available from the corresponding author on reasonable request.

## Abbreviations

BBC: Bacterial Blight of Cotton
*Xcm*: *Xanthomonas citri pv. malvacearum*
TALE: Transcription Activator-Like Effector
*tal*: transcription-activator like
RFLP: Restriction Fragment Length Polymorphism
CRR: Central Repeat Region
RVDs: Repeat Variable Diresidues
EBE: Effector Binding Element
T3SE: Type III Secreted Effector
NLS: Nuclear Localization Signals
AD: Activation Domain
M: mutant
HR: Hypersensitive Response
Km: Kanamycin
