# Supplementary material for: Identification of a virulence tal gene in the cotton pathogen, Xanthomonas citri pv. malvacearum strain Xss-V2–18
Source: BMC Microbiol. 2020 Apr 15;20:91. doi: 10.1186/s12866-020-01783-x (PMC7160923; doi:10.1186/s12866-020-01783-x)
Supplement: Supplementary file 1 — Additional file 1: Table S1. Primers used in this study. Figure S1. Predicted theoretical target site logo. (A) Target site logo for Tal2 of Xss-V2-18. (B) Target site logo for Tal6 of MSCT1 and Tal26 of MS14003. Based on TALgetter (Galaxy v1.1 http://galaxy.informatik.uni-halle.de/) [file 12866_2020_1783_MOESM1_ESM.docx]

**Identification of a virulence *tal* gene in the cotton pathogen, *Xanthomonas citri* pv. *malvacearum* strain Xss-V_2_-18**

Fazal Haq^1,2^, Shiwang Xie^1^, Kunxuan Huang^1,2^, Syed Mashab Ali Shah^1,2^, Wenxiu Ma^1,2^, LuLu Cai^1,2^, Xiameng Xu^1,2^, Zhengyin Xu^1,2^, Sai Wang^1^, Lifang Zou^1,2^, Bo Zhu^1^ and Gongyou Chen^1,2*^

^1^School of Agriculture and Biology, Shanghai Jiao Tong University/Key Laboratory of Urban Agriculture by the Ministry of Agriculture, Shanghai, 200240, China

^2^State Key laboratory of Microbial Metabolism, School of life Science and Biotechnology, Shanghai Jiao Tong University, Shanghai, 200240, China

**^*^Correspondence:** [gyouchen@sjtu.edu.cn](mailto:gyouchen@sjtu.edu.cn)

**Running title:** Tal2 contributes to *Xcm* virulence

**Table S1.** Primers used in this study

| **Primer** | **Sequence (5’→3’; restriction sites underlined)** | **Description** |
| --- | --- | --- |
| pKMSA1-5F | GGACCCGGGGTAGGGACCACAGACCGCTAG | Amplifies a 580-bp fragment (designated *a*) upstream of the *talB* cluster in Xcc049; used for deletion mutagenesis of *Xcm* Xss-V2-18 |
| pKMSA1-5R | CCAAAGCTTACTGTCGAACGCACCTTCGGT |  |
| pKMSA1-3F | TGGAAGCTTGACCTTGATGCGCCTAGCC | Amplifies a 350-bp fragment (designated *b*) downstream of the *talB* cluster of Xcc049; used for deletion mutagenesis of *Xcm* Xss-V2-18 |
| pKMSA1-3R | TCCTCTAGACTGAGGCAATAGCTCCATC |  |
| pKMSA2-5F | GGACCCGGGGCGCACGCGCCCTGGAGGCCTT | Amplifies a 150-bp fragment *c* upstream of the *talC* cluster of Xcc049; used for deletion mutagenesis of *Xcm* Xss-V2-18 |
| pKMSA2-5R | CCAAAGCTTCCGTCAGTGCATTGCGCCAT |  |
| pKMSA2-3F | TGGAAGCTTTTGACCAACGACCACCTCGTCG | Amplifies a 300-bp fragment *d* downstream of the *talC* cluster of Xcc049; used for deletion mutagenesis of *Xcm* Xss-V2-18 |
| pKMSA2-3R | TCCTCTAGAACTGCGGGCTTCGAGTTCGGTG |  |
| FP | ACCTACAACAAAGCTCTCATCAACC | Tn*5* forward primer |
| RP | GCAATGTAACATCAGAGATTTTGAG | Tn*5* reverse primer |
| *tal*-F | GGAGAGTTGAGAGGTCCACCGTTAC | *tal* forward primer at N-terminal before repeat region |
| *tal*-R | CGGGAATACGGCGATTGGTTCTTTT | *tal* reverse primer at C-terminal after repeat region |

**
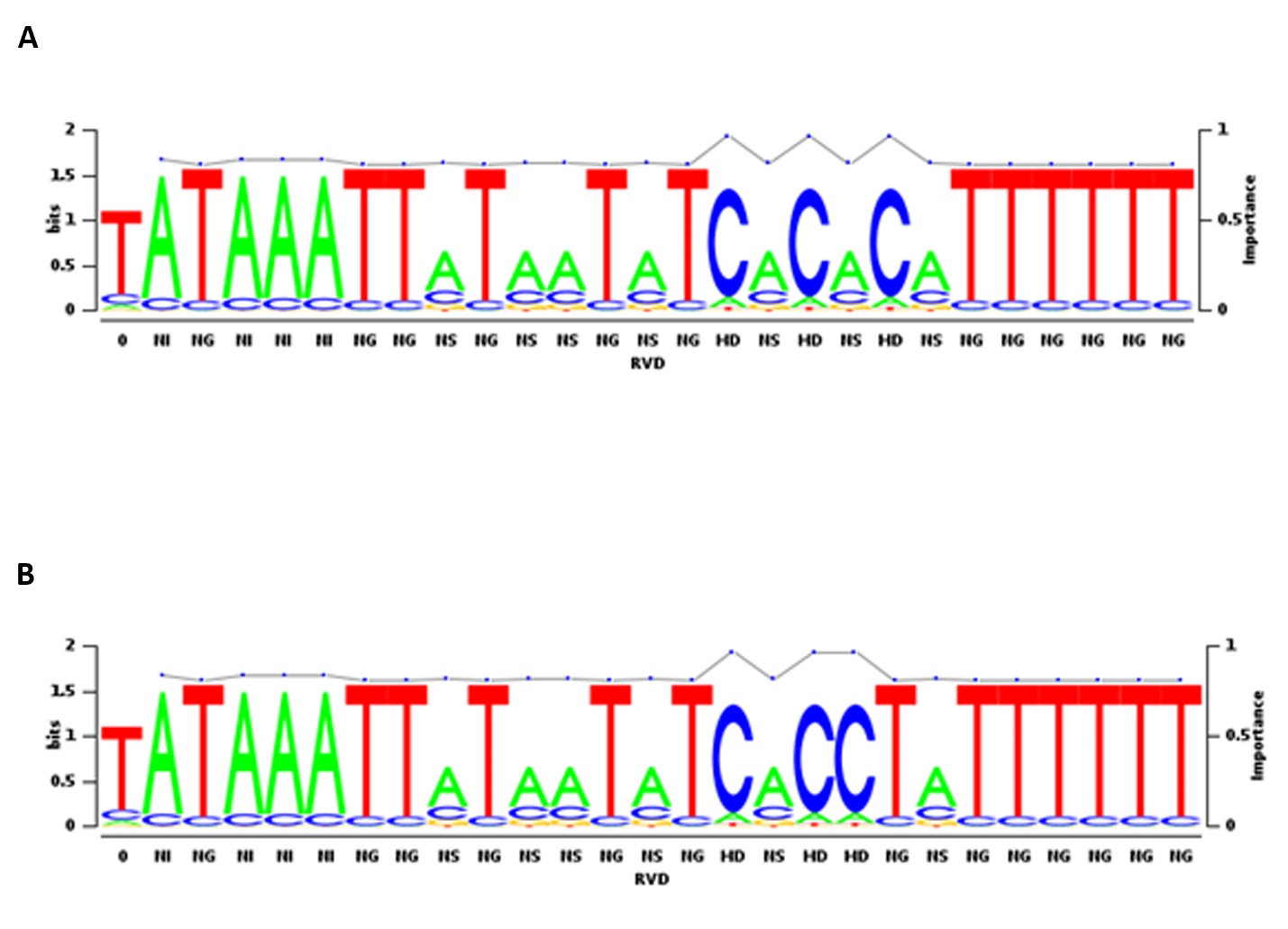
**

**Fig. S1.** Predicted theoretical target site logo. **(A)** Target site logo for Tal2 of Xss-V_2_-18. **(B)** Target site logo for Tal6 of MSCT1 and Tal26 of MS14003. Based on TALgetter (Galaxy v1.1 <http://galaxy.informatik.uni-halle.de/>)
